# Glutathione S-Transferase Mu-3 Predicts a Better Prognosis and Inhibits Malignant Behavior and Glycolysis in Pancreatic Cancer

**DOI:** 10.3389/fonc.2020.01539

**Published:** 2020-08-28

**Authors:** Shunda Wang, Jinshou Yang, Cheng Ding, Junjie Li, Lei You, Menghua Dai, Yupei Zhao

**Affiliations:** ^1^Department of General Surgery, Peking Union Medical College Hospital, Chinese Academy of Medical Sciences and Peking Union Medical College, Beijing, China; ^2^Department of Pathology, Peking Union Medical College Hospital, Chinese Academy of Medical Sciences and Peking Union Medical College, Beijing, China

**Keywords:** glutathione S-transferase mu-3, pancreatic cancer, proliferation, ROS, glycolysis

## Abstract

**Background:** Pancreatic cancer (PC) is a lethal malignancy with an extremely unfavorable 5-year survival rate and a high mortality rate. Glutathione S-transferase mu-3 (GSTM3) has been shown to exert different functions in the progression and development of various cancers, except for PC. This study aimed to explore the role of GSTM3 in the malignant behavior and metabolic aspects of PC, its clinical significance, and its possible molecular mechanism in pancreatic cancer.

**Methods:** Tumor microarrays of pancreatic ductal adenocarcinoma (PDAC) were used to evaluate the clinicopathological variables and GSTM3 expression by immunohistochemical staining. Kaplan–Meier survival and Cox regression analyses were further performed to assess the prognosis. The effect of GSTM3 on PC aggressiveness was detected using overexpressing and silencing transfection methods. Western blot, RT-qPCR, CCK-8, and cell cycle assay were applied to evaluate the expression level and proliferation. A xenograft animal model was assessed. Reactive oxygen species (ROS) were measured using the laser confocal scanner and glycolysis was detected using an Agilent Seahorse kit. RNA sequencing was used to assess the underlying mechanism and the signaling pathway involved.

**Results:** GSTM3 was relatively poorly expressed in PDAC tissues compared to para-tumoral tissues and a high level of GSTM3 indicated good overall survival. Functionally, overexpression of GSTM3 could significantly inhibit cell proliferation by delaying the G0/G1 transition, whereas the opposite results were found in the GSTM3 downregulation group. In addition, xenograft animal models further confirmed the effect on proliferation. Moreover, silencing of GSTM3 induced ROS accumulation and promoted glycolysis in PC, indicating its tumor suppressive effect, and vice versa when GSTM3 was upregulated. Finally, RNA sequencing results demonstrated that GSTM3 facilitates anti-tumorigenicity partly via the JAK-STAT signaling pathway in PC.

**Conclusion:** GSTM3 inhibited tumor progression and altered the metabolic pattern in PC. This may be a potential predictive biomarker in PC and a prospective therapeutic target.

## Introduction

Pancreatic cancer (PC) is one of the most lethal malignant tumors with a poor prognosis and a high mortality rate (1). Pancreatic ductal adenocarcinoma (PDAC) makes up a majority of PCs. It is difficult to make an early diagnosis, with a 5-year overall survival (OS) rate of <10% in recent decades (2, 3); 80–85% of patients lack diagnostic symptoms at the early stage and therefore miss the best opportunity for operation, leading to cancer progression or distant metastasis (4). Although chemotherapy based on gemcitabine has proved effective in some patients, chemoresistance, and its adverse effects also impair the OS (5). Therefore, elucidation of the molecular mechanism underlying tumor progression is extremely essential for improving outcomes among patients with PC.

The glutathione S-transferase (GST) family, the primary phase II metabolic enzymes involved in the detoxification of xenobiotic compounds and clearance of reactive oxygen species (ROS), includes eight distinct classes: mu, pi, sigma, alpha, omega, theta, kappa, and zeta (6–8). The GSTM3 gene, which encodes the mu class 3 of enzymes, is located on chromosome 1p13.3 and has been investigated in various malignancies (9, 10). Particularly, GSTM3 has been shown to be dysregulated in various cancers such as renal cancer (11), prostate carcinoma (12), breast carcinoma (13), hepatic carcinoma (14), colorectal carcinoma (15), and cervical malignancy (16). To the best of our knowledge, there is no consensus regarding whether it acts as a tumor suppressor or promoter. The exact role of GSTM3 in PDAC remains unclear and the present study is the first to explore the clinical significance and biological role of GSTM3 in PC.

Herein, we assessed the role of GSTM3 in PC and investigated its possible association with the prognosis of PDAC patients. We also explored the effects of GSTM3 alteration on tumor progression and metabolism of PC and the possible underlying mechanism.

## Methods

### Clinical Specimens and Immunohistochemistry

A total of 97 PDAC tissues, including cancerous and adjacent tissues were obtained from Peking Union Medical College Hospital (PUMCH). The tissue microarrays (TMAs) were constructed as described previously (17). The 8th edition of the TNM staging system put forth by the American Joint Committee on Cancer (AJCC) was applied (18). Follow-up was conducted via telephone interviews or review of the medical records. The OS was assessed as the period between the date of diagnosis and the last date at which patients were known to be alive or the date of death. The study got approval from the ethics committee of PUMCH. Patients written informed consent prior to inclusion in the present study. Immunohistochemistry (IHC) was performed using rabbit anti-human GSTM3 polyclonal antibodies (1:200; 15214-1-AP, Proteintech). The *H*-score, a combination of the proportion of positive cells and the staining intensity, was applied to assess the results of IHC. The *H*-score ranged from 0-300 and the optimal cutoff value for it was defined via the largest Youden index under the receiver operating characteristic (ROC) (19). Two pathologists blinded to the clinicopathological information evaluated the staining results independently. Each TMA was divided into five parts and the average value represented the result of the patient.

### Cell Culture

PC cell lines (MIA PaCa-2, PANC-1, CFPAC-1, T3M4, BxPC-3, and AsPC-1) were obtained from the American Type Culture Collection (Virginia, USA). They were cultured, respectively in Dulbecco's Modified Eagle Medium, Iscove's Modified Dulbecco's Medium, and Roswell Park Memorial Institute 1640 medium (Hyclone) with 10% fetal bovine serum (Gibco) under incubation at 37°C with 5% carbon dioxide.

### Transfection

PANC-1 and MIA PaCa-2 cells were seeded and cultured until the confluence reached 60%. Then, they were transfected with GSTM3 knockdown lentivirus (shGSTM3), scramble control lentivirus (shNC), GSTM3 overexpressing lentivirus (GSTM3-OE), and negative control (NC) lentivirus (GSTM3-NC) following the manufacturer's instructions. Lentivirus constructions of *GSTM3* knockdown and overexpression were obtained from Gene Pharma (Shanghai, China). Stable transfection cell lines were screened with 1 μg/ml of puromycin for more than 14 days. Monoclonal isolation was performed. Quantitative real-time polymerase chain reaction (qRT-PCR) and western blot assays were adopted to validate the transfection efficiency.

### RNA Isolation and qRT-PCR

RNA was extracted from PC cells using TRIzol reagent (Ambion, Life Technologies). PrimeScript^TM^ RT Master Mix and the SYBR Green PCR Kit (TaKaRa, Japan) were used for reverse transcription and qRT-PCR assays. PCR assays were performed using StepOnePlus™ (Applied Biosystems, California, USA). We used the GSTM3 forward primer GGAGGCAAGGGACGGAGA and reverse primer TTCCGAGCCTTCGAGGACTAG. β-actin forward, TGAAGGTAGTTTCGTGGATGC; β-actin reverse, TCCCTGGAGAAGAGCTACGA.

### Western Blot

Cells were lysed using the RIPA lysis buffers system combined with a proteinase inhibitor (Applygen, China). The protein concentration was calculated using the bicinchoninic acid (BCA) Kit (Thermo Fisher Scientific, USA). Approximately 20.0 μg of the lysate was added into 12% SDS-PAGE gel and then ran for 2.5 h at 80 V. Immobilon-PVDF membranes (Millipore, USA) was used for immunoblotting. Protein was transferred to the membrane at 400 mA for 1.5 h on ice. After milk blocking, the membranes were probed with the following primary antibodies: anti-GSTM3 (1:1000; Proteintech), anti-β-actin (Abcam, USA), and anti-GAPDH (Santa Cruz, USA). In addition, we used the following antibodies: LDHB (14824– 1-AP, Proteintech), anti-JAK2, anti-p-JAK2, anti-STAT4, anti-p-STAT4, anti-HK2, anti-PKM2, and anti-cyclin A2, B1, and E1 (Cell Signaling Technology, USA). Next, the membrane was incubated with secondary antibody (horseradish peroxidase-labeled) (Lablead, China) for 2 h in 5% non-fat milk. The protein was visualized by exposing the enhanced chemiluminescence system to X-ray (Tanon 5500; Tanon Inc., Shanghai, China).

### Immunofluorescence

Paraffin sections were dewaxed, dehydrated in gradient alcohol, and then rinsed with 0.01 M phosphate buffered saline with Tween for 5 min. After locking with 10% bovine serum albumin, they were incubated with anti-GSTM3 antibody (1:200; ab74749, Abcam). Then, they were rinsed with phosphate buffered saline (PBS), sealed with buffered glycerol, and finally observed under fluorescence microscopy.

### Cell Counting Kit-8 (CCK-8) Assay

According to the manufacturer's instructions, the CCK-8 kit (Dojindo, Japan) was used to detect the cell proliferation. Cells were plated into 96-well plates at 3 × 103 cells/well. Then, 10 μl of the reagent was added 0, 24, 48, 72, 96, and 120 h later. Absorbance was calculated at an optical density (OD) of 450–630 nm after an additional 2 h of CCK-8 incubation at 37°C using the microplate reader (Thermo Labsystems, Finland).

### Cell Cycle Assay

When the confluence reached ~70–80%, cells (1 × 10^6^/ml) were collected and fixed in ethanol (70%) at a temperature of −20°C for more than 18 h. Cells were washed with PBS and centrifuged simultaneously at 1000 rpm. Then, the cells were incubated with RNase A. After adding propidium iodide to the cellular suspension, they were assessed by flow cytometry (BD Accuri™ C6 Plus, USA). Finally, important molecules related to the cell cycle were assessed via western blot assays.

### Reactive Oxygen Species Measurements

The activity of ROS was detected using the DCFH-DA kit (S0033, Beyotime) following the manufacturer's guidebook. 2′,7′-Dichlorodihydrofluorescein diacetate was used as the oxidation-sensitive fluorescent probe. PC cells (1-5 × 10^6^/ml) were incubated with a probe at 37°C for 20 min in a 35 mm porous glass bottom dish (Cellvis, USA) and the nuclei were stained using Hoechst (Beyotime). A laser scanning confocal microscope (Nikon, Japan) was used to capture the image. Quantitative DCF fluorescence was measured by taking the average of measurements obtained on multiple views.

### Metabolism Experiments

The XF96 Extracellular Flux Analyzer (Seahorse Bioscience, USA) was used to detect the extracellular acidification rate (ECAR) and the proton efflux rate (PER) in PC cells. Briefly, cell suspensions of PANC-1 and MIA PaCa-2 cells were seeded into the XF96 cell culture microplate (Seahorse Bioscience, USA) at a density of 3 × 10^4^ cells prior to the experiment. Cells were incubated with base assay medium. The glycolytic rate assay was performed using the Agilent Seahorse XF Glycolytic Rate Assay Kit (103344–100; Agilent Technologies). Finally, a BCA kit (Beyotime) was adopted to calculate the concentration of protein for normalization of the results at the end of the experiments.

### RNA Sequence

Total RNA was extracted using TRIzol. Then, nanodrop for OD_260/280_ and Agilent 2100 for length segments were performed. RNA libraries were constructed according to the NEBNext Ultra RNA Library Prep Kit (NEB, USA) and further sequenced on an Illumina HiSeq/MiSeq 4000 with 100 million total reads per sample (50 million paired reads). After the quality control of the sequencing data was passed, all data were analyzed according to the manufacturers' instructions. Differentially expressed genes were then identified by a fold change >2. Enrichment analysis was performed.

### Animal Xenograft Experiments

Female BALB/c nude mice (4 weeks old) were randomly classified to two sets and maintained in pathogen-free environments. Each experimental group included four mice. Panc-1 cells (5 × 106/ml) transfected with shGSTM3 or shNC were injected into the groin of the mice with PBS (100 μl suspension) subcutaneously. The formula 1/2 × length × width^2^ was applied to calculate the tumor volume. And tumors were weighed every week. Animals were euthanized 7 weeks later. Tumors were harvested, weighed, and assessed via IHC. Animal studies were performed in keeping with the principles put forth by the Experimental Animals Management Committee.

### Statistical Analysis

Each experiment was performed at least three times. Continuous data was calculated using either the student's *t*-test or Mann Whitney *U*-test. Categorical variables were analyzed by either Fisher's test or Pearson's chi-squared test. Univariate and multivariate analyses were performed using the Kaplan–Meier method or Cox proportional hazards regression. Data are presented as the mean ± standard deviation. Statistical analyses were performed by SPSS23.0 (SPSS Inc., Chicago, USA) and GraphPad Prism 7 (GraphPad Software, San Diego, USA). Analysis items with *P* values < 0.05 were considered statistically significant.

## Results

### GSTM3 Expression in PDAC Samples and Its Association With a Good Prognosis

**Figure 1:**
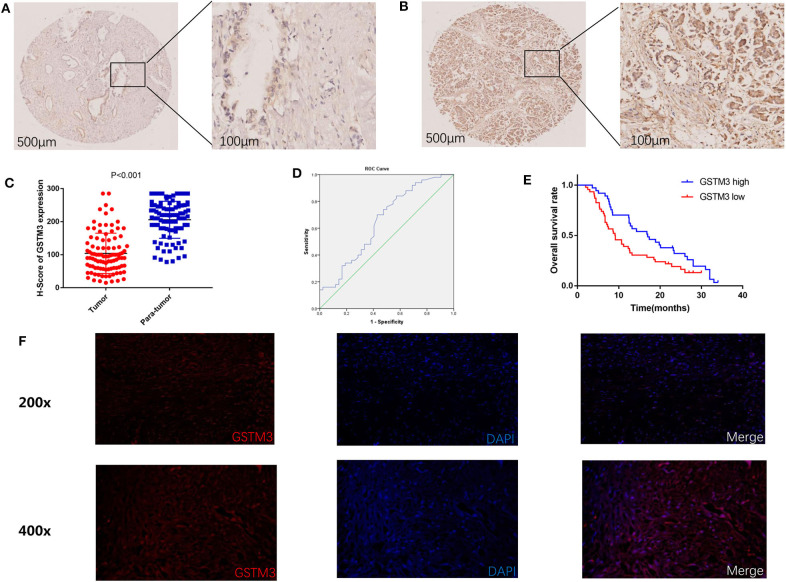
Immunohistochemical results of GSTM3 expression in pancreatic cancer tissues and Kaplan–Meier curve of overall survival according to the level of GSTM3. **(A)** Representative images of GSTM3 staining in tumor tissues by immunohistochemistry staining. Scale bar, 500, 100 μm. **(B)** Representative images of GSTM3 staining in para-tumor tissues by immunohistochemistry staining. Scale bar, 500, 100 μm. **(C)** Comparison of *H*-scores for GSTM3 level between tumor and non-tumor tissues (*p* < 0.001, Mann-Whitney *U*-test). **(D)** ROC curve for tumoral *H*-scores of GSTM3 for survival status. **(E)** Kaplan–Meier survival curve of the effect of tumoral GSTM3 expression on overall survival of pancreatic cancer patients (*P* = 0.034; log-rank test). **(F)** Tissue immunofluorescence of pancreatic cancer cells showed the cytoplasmic location of GSTM3. Original magnification, ×200 (upper panels) or ×400 (lower panels).

In our study, IHC was used to detect the level of GSTM3 expression in 97 samples of cancerous tissues and matched para-cancerous tissues. Positive staining for GSTM3 was detected in the cytoplasm of cancer cells (Figures 1A,B). Tissue immunofluorescence of PC also showed the cytoplasmic location of GSTM3 (Figure 1F). The level of GSTM3 expression was significantly lower in cancerous tissues compared to para-cancerous tissues (Figure 1C). The cutoff value of *H*-score was determined as the largest Youden index after analyzing the ROC curve (68.5) (Figure 1D). The cases with an *H*-score less than the cut-off value were regarded as low GSTM3 expression, while the others were high GSTM3 expression. The clinicopathological parameters of all the patients are summarized in Table 1. Statistical analyses revealed no significant association between the expression level of GSTM3 and clinicopathological characteristics.

**Table 1:** Correlations of GSTM3 expression in tissues and clinicopathological parameters.

**Variables**	**Number (n)**	**GSTM3 expression**		***P* value**
		**High group**	**Low group**	
**Gender**				0.138
Male	62	24	38	
Female	35	19	16	
**Age, y**				0.105
≥60	54	20	34	
<60	43	23	20	
**CA19-9**				0.533
Elevated	67	28	39	
Normal	18	9	9	
**T**				0.889
T1-2	29	13	16	
>T3	67	29	38	
**N**				0.623
N0	53	22	31	
N1	43	20	23	
**AJCC stage**				0.520
I-IIa	47	19	28	
≥IIb	49	23	26	
**Tumor size**				0.533
>4	42	20	22	
≤4	51	21	30	
**Locations**				0.410
Head	54	26	28	
Body-tail	38	15	23	
**Differential degree**				0.403
High/moderate	71	34	37	
Low	14	5	9	
**Perineural invasion**				0.448
No	38	15	23	
Yes	57	27	30	
**Vascular invasion**				0.328
No	66	27	39	
Yes	29	15	14	

*P value in bold is statistically significant. Total patient number does not equal 97 in all categories, as patient information was not available for all cases. T, tumor; N, lymph node; AJCC, American Joint Committee on Cancer; GSTM3, Glutathione S-transferase mu-3*.

Next, we assessed the association between GSTM3 level and patient prognosis. Fourteen patients were lost to follow-up and eventually the data of 83 patients were analyzed. Kaplan–Meier analysis revealed that patients with high GSTM3 levels experienced a longer OS than patients with low GSTM3 levels (*P* = 0.034) (Figure 1E). Age, T stage, AJCC stage, vascular invasion were also prognostic indicators in the univariate analysis (*P* < 0.05; Table 2). Finally, multivariable Cox regression analysis revealed that GSTM3 was a risk factor for OS among patients with PC (hazard ratio [HR] = 1.208, 95% confidence interval [CI]: 1.022–1.608, *P* = 0.012) (Table 2). Taken together, our results proved that the level of GSTM3 expression might be an independent predictor of the OS.

**Table 2:** Univariate and multivariate analyses for prognosis factors in pancreatic cancer patients.

**Variables**	**Number (*n*)**	**Univariate analysis**			**Multivariate analysis**		
		**OS (Median ± SE, m)**	**95% CI**	***P* value**	**HR**	**95% CI**	***P* value**
**Gender**				0.073			
Male	57	12.30 ± 1.877	8.601–15.999				
Female	26	12.50 ± 1.976	8.627–16.373				
**Age, y**				**0.046**			
≥60	46	9.20 ± 1.242	6.765–11.635				
<60	37	13.70 ± 0.912	11.912–15.488				
**CA19-9**				0.125			
Elevated	57	9.20 ± 1.456	6.347–12.053				
Normal	18	16.80 ± 2.192	12.504–21.096				
**T stage**				**0.002**			**0.001**
T1-2	26	19.60 ± 2.74	14.228–24.972		0.380	0.219-0.660	
>T3	56	9.20 ± 1.309	6.635–11.765		1		
**N stage**				0.077			
N0	48	12.80 ± 3.11	6.698–18.911				
N1	34	8.70 ± 2.77	3.271–14.129				
**AJCC stage**				**0.032**			
I-IIa	42	14.30 ± 3.34	7.737–20.863				
≥IIb	40	9.10 ± 1.66	5.846–12.354				
**Tumor size**				0.054			
>4	35	9.20 ± 2.021	5.240–13.160				
≤4	44	13.20 ± 3.372	6.591–19.809				
**Locations**				0.776			
Head	48	12.40 ± 1.155	10.137–14.663				
Body-tail	30	12.50 ± 1.712	9.145–15.855				
**Differential degree**				0.404			
High/moderate	58	12.70 ± 0.435	11.847–13.553				
Low	13	9.20 ± 1.11	7.023–11.377				
**Perineural invasion**				0.155			
No	32	13.20 ± 3.606	6.132–20.268				
Yes	49	11.20 ± 1.983	7.313–15.087				
**Vascular invasion**				**0.030**			
No	55	13.70 ± 3.231	7.368–20.032				
Yes	26	9.20 ± 1.402	6.452–11.948				
**GSTM3 expression**				**0.034**			**0.012**
High	37	14.00 ± 2.43	9.233–18.767		1.208	1.022-1.608	
Low	46	9.10 ± 1.55	6.062–12.138		1		

*P value in bold is statistically significant. T, tumor; N, lymph node; AJCC, American Joint Committee on Cancer; GSTM3, Glutathione S-transferase mu-3; OS, overall survival; HR, hazard ratio; 95% CI, 95% confidence interval*.

### PANC-1 and MIA PaCa-2 Cells Were Selected for Further Investigation

**Figure 2:**
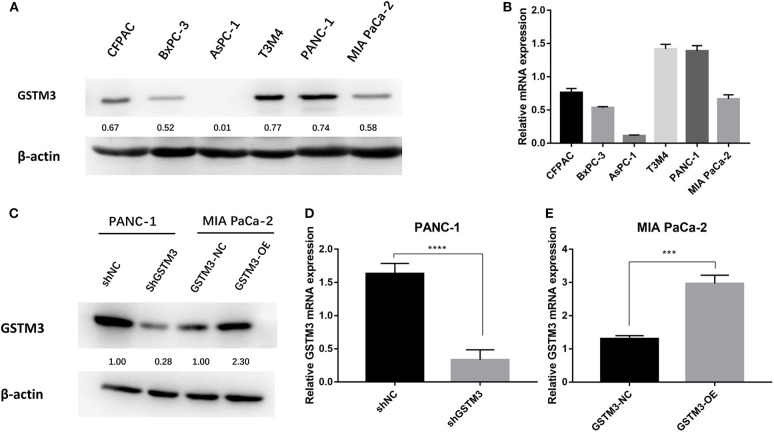
GSTM3 expression in six pancreatic cancer cell lines and PANC-1 and MIA PaCa-2 were selected for further analysis. **(A)** The protein level of GSTM3 as assessed by western blot. **(B)** The RNA level of GSTM3 as assessed by PCR. **(C)** The efficiency of GSTM3 knockdown and GSTM3 overexpression were confirmed by western blot. **(D,E)** The efficiency of GSTM3 knockdown and GSTM3 overexpression were validated by qRT-PCR. Data are presented as mean ± SD. (Student's *t*-test; ***p < 0.001; ****p < 0.0001) GSTM3, Glutathione S-transferase mu-3; GSTM3-OE, GSTM3 overexpression; GSTM3-NC, GSTM3 negative control; shGSTM3, GSTM3 knockdown; shNC, scramble control (*n* = 3).

GSTM3 expression was explored in six PC cell lines by qRT-PCR assay and western blot. Specifically, we found that PANC-1 cells had relatively higher levels of GSTM3 expression, whereas MIA PaCa-2 cells had relatively lower levels (Figure 2A). The qRT-PCR assay revealed that the GSTM3 mRNA level corresponded to its protein expression (Figure 2B). Based on these results, MIA PaCa-2 and PANC-1 cells were chosen for further investigation. As described in the methods section, stable transfections of GSTM3 knockdown in PANC-1 cells and GSTM3 overexpression in MIA PaCa-2 cells were established using lentivirus vectors. The efficiency of GSTM3 knockdown and GSTM3 overexpression compared to the NC cell lines were confirmed using qRT-PCR and western blot (Figures 2C–E).

### GSTM3 Inhibited PC Proliferation *in vitro*

**Figure 3:**
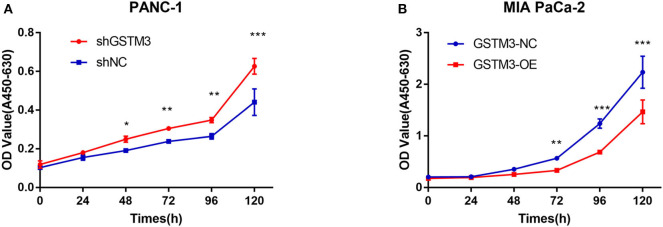
GSTM3 inhibited cell proliferation in pancreatic cancer. **(A)** Cell viability (represented by A450-630 value) of PANC-1 cells treated with shGSTM3 was detected using the cell counting kit-8 assay. Error bars represent the standard deviations. **(B)** Cell viability (represented by A450-630 value) of MIA PaCa-2 cells treated with GSTM3-OE was detected using the cell counting kit-8 assay. Error bars represent the standard deviation. Data are presented as mean ± SD. (Student's *t*-test; *P < 0.05; **p < 0.01; ***p < 0.001) GSTM3, Glutathione S-transferase mu-3; GSTM3-OE, GSTM3 overexpression; GSTM3-NC, GSTM3 negative control; shGSTM3, GSTM3 knockdown; shNC, scramble control (*n* = 3).

CCK-8 assay was applied to investigate the role of GSTM3 in proliferation. Downregulation of GSTM3 increased the cell proliferation capacity compared to shNC in PANC-1 stable cell lines (Figure 3A). Also, the proliferation assay revealed that GSTM3 overexpression in MIA PaCa-2 stable cell lines resulted in significantly abolished proliferation capacity compared to NC cells (Figure 3B).

### GSTM3 Hampered PC Proliferation by Enriching G0/G1 Retention

**Figure 4:**
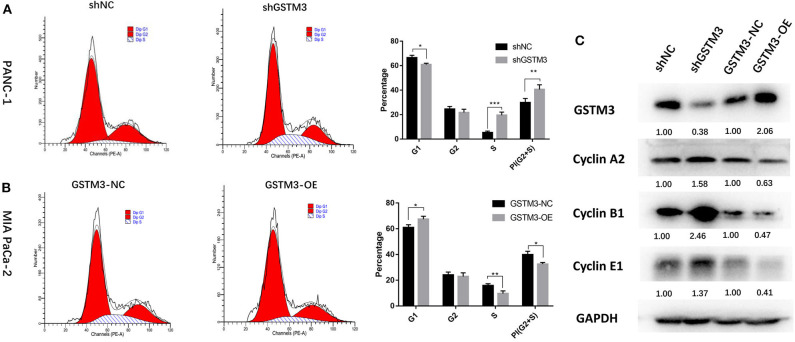
Cell cycle assay showed that GSTM3 hampered proliferation of pancreatic cancer cells through the enrichment of G0/G1 retention. **(A,B)** Cell cycle was detected using flow cytometry and the proportions of G1, G2, and S phases were analyzed. **(C)** The key components expressed in the cell cycle were evaluated by western blot. Data are presented as mean ± SD. (Student's *t*-test; *P < 0.05; **p < 0.01; ***p < 0.001) GSTM3, Glutathione S-transferase mu-3; GSTM3-OE, GSTM3 overexpression; GSTM3-NC, GSTM3 negative control; shGSTM3, GSTM3 knockdown; shNC, scramble control (*n* = 3).

To explore the effects of GSTM3 on the cell cycle of PC cells, we performed flow cytometry and detected changes in cyclin protein induced by alteration of GSTM3 in MIA PaCa-2 and PANC-1 cells. First, GSTM3 downregulation led to an decrease in the number of cells in the G0/G1 phase and an increase in the number of cells in the G2/M and S phases, resulting in a higher proliferation index. Additionally, opposite results were produced in MIA PaCa-2 cells with GSTM3-OE (Figures 4A,B). Our results implied that GSTM3 might impede the transition of G1/S phase. Simultaneously, western blot was used to detect the crucial proteins related to the cell cycle. The results showed that the levels of the G1/S marker (cyclin E1) and G2/M markers (cyclin A2 and cyclin B1) were reduced when GSTM3 was upregulated and were increased when GSTM3 was downregulated (Figure 4C).

### GSTM3 Inhibited PC Cell Proliferation *in vivo*

**Figure 5:**
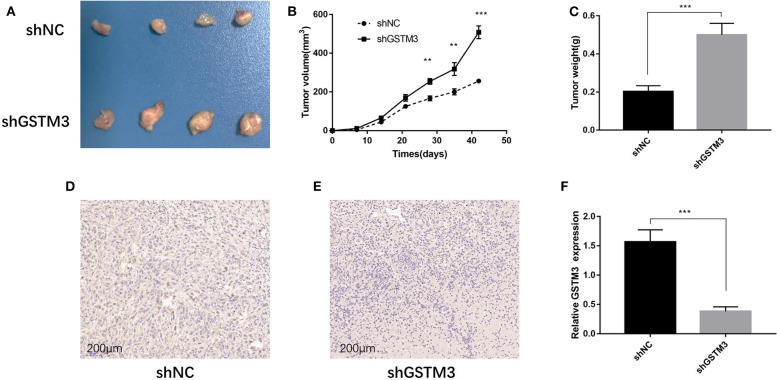
Knockdown of GSTM3 promoted pancreatic cancer cell growth *in vivo*. **(A)** Tumors were photographed after harvesting from nude mice inoculated with PANC-1 cells transfected with shGSTM3 and a negative control. **(B)** The tumors generated from cells with low GSTM3 level were significantly larger than those generated from control cells 6 weeks after injection. **(C)** The tumors in the shGSTM3 group were significantly heavier than those in the control group. **(D,E)** Immunohistochemical staining of GSTM3 in xenograft tumor tissue obtained from mice. Scale bar, 200 μm. **(F)** Real-time PCR was used to confirm the expression levels of GSTM3 in xenograft tumors. Data are presented as mean ± SD. (Student's *t*-test; **p < 0.01; ***p < 0.001) GSTM3, Glutathione S-transferase mu-3; GSTM3-OE, GSTM3 overexpression; GSTM3-NC, GSTM3 negative control; shGSTM3, GSTM3 knockdown; shNC, scramble control (*n* = 3).

The xenograft tumor model was applied to explore the effect of GSTM3 on cell proliferation *in vivo*. PANC-1 cells stably transfected with shGSTM3 and shNC were injected into nude mice subcutaneously. After observation for 6 weeks, the results showed that GSTM3 knockdown promoted tumor growth compared to the shNC group (Figure 5A). Also, tumor volumes and weights in the shGSTM3 group were remarkably higher than that in the shNC group (Figures 5B,C). IHC staining revealed that the GSTM3 level in the xenograft tumors were lower in the shGSTM3 group than that in the shNC group (Figures 5D,E). RT-qPCR assay further demonstrated that the level of GSTM3 was reduced in the xenograft tumors of the shGSTM3 group (Figure 5F). In summary, this result showed that GSTM3 is remarkably correlated with the proliferation capacity of PDAC and exerted an important role in PDAC progression.

### GSTM3 Reduced the Activity of ROS

**Figure 6:**
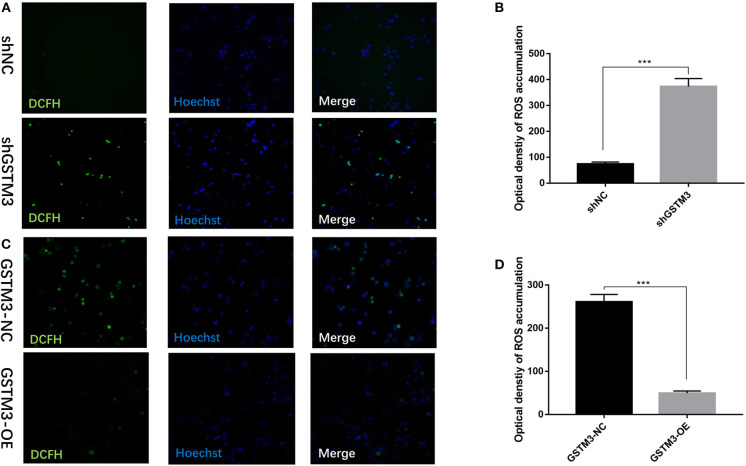
GSTM3 inhibited reactive oxygen species (ROS) activity in pancreatic cancer cells. **(A)** Representative confocal micrographs of ROS in shNC and shGSTM3 cells incubated with DCFH-DA. **(B)** Quantitative analysis of ROS accumulation in the shNC and shGSTM3 groups. **(C)** Representative confocal micrographs of ROS in GSTM3-NC and GSTM3-OE cells incubated with DCFH-DA. **(D)** Quantitative analysis of ROS accumulation in the GSTM3-NC and GSTM3-OE groups. Data are presented as mean ± SD. (Student's *t*-test; ***p < 0.001) GSTM3, Glutathione S-transferase mu-3; GSTM3-OE, GSTM3 overexpression; GSTM3-NC, GSTM3 negative control; shGSTM3, GSTM3 knockdown; shNC, scramble control (*n* = 3).

To investigate the biological effects of GSTM3 on ROS, we measured the activity of ROS in PC cells by the loss- or gain-of-function strategies to knockdown or overexpress GSTM3 in PC cells. The activity of ROS was significantly increased in the GSTM3 knockdown group compared to the NC group (Figures 6A,B). When GSTM3 was upregulated, the activity of ROS was obviously reduced than that in the normal group (Figures 6C,D). This result suggested that GSTM3 expression indeed reduced ROS activity *in vitro*.

### GSTM3 Inhibited Glycolysis in Pancreatic Cancer Cells

**Figure 7:**
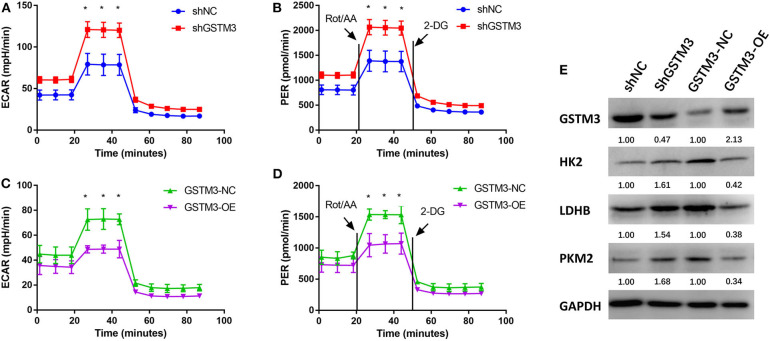
GSTM3 inhibited glycolysis in pancreatic cancer cells. **(A,B)** GSTM3 knockdown significantly increased the ECAR and also increased the glycolysis PER in PANC-1 cells. **(C,D)** GSTM3 overexpression remarkably decreased the ECAR and also decreased glycolysis PER in MIA PaCa-2 cells. **(E)** The key enzymes in glycolysis (HK2, LDHB, and PKM2) were evaluated by western blot. Data are presented as mean ± SD. (Student's *t*-test; *p < 0.05) GSTM3, Glutathione S-transferase mu-3; GSTM3-OE, GSTM3 overexpression; GSTM3-NC, GSTM3 negative control; shGSTM3, GSTM3 knockdown; shNC, scramble control (*n* = 3).

The ECAR and PER were assessed using the XF96 Extracellular Flux Analyzer. The ECAR was increased among cells in the GSTM3 knockdown group but decreased among cells in the GSTM3 overexpression group. In addition, the PER was also significantly increased in the shGSTM3 group than in the shNC group for PANC-1 cells. On the contrary, the PER was decreased in the GSTM3-OE group than in the GSTM3-NC group for MIA PaCa-2 cells (Figures 7A–D). The above results indicated that GSTM3 altered cellular glycolysis in PC cells. Further, we evaluated key enzymes related to glycolysis by western blot. The results manifested that shGSTM3 enhanced the expression of HK2, PKM2, and LDHB (crucial enzymes that enhance the glycolysis) and vice versa in the GSTM3-OE group (Figure 7E).

### RNA Sequence and Mechanism

**Figure 8:**
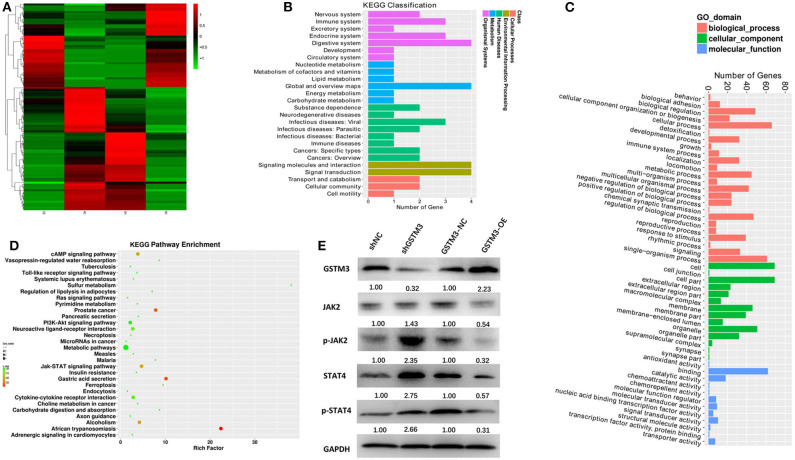
Bioinformatic analysis of the results of RNA sequencing and western blot revealed the mechanism underlying the role of GSTM3 in pancreatic cancer. **(A)** Heatmap of gene transcription profiles that were negatively or positively regulated by GSTM3 in pancreatic cancer cells. **(B)** KEGG classification of differentially expressed mRNA. **(C)** GO enrichment analyses of differentially expressed genes. **(D)** KEGG pathway enrichment of differentially expressed genes. **(E)** Western blot analysis detected the protein levels of JAK2,p-JAK2, STAT-4, and p-STAT-4 in the context of depletion or enrichment of GSTM3.

The mRNA expression profiling was performed among both GSTM3 overexpression and GSTM3 knockdown cells. The heatmap showed the intersection of genes in the two groups (Figure 8A). Kyoto Encyclopedia of Genes and Genomes (KEGG) and Gene Ontology classification of differentially expressed genes (false discovery rate < 0.05, *P* < 0.001) were performed (Figures 8B,C). These genes regulated by GSTM3 were mainly focused on metabolism, antioxidant activity and signal transduction. Moreover, the JAK-STAT pathway was enriched in the KEGG pathway analysis (Figure 8D). Further validation by western blot revealed that the level of JAK2 and STAT4 in the shGSTM3 were enhanced significantly than that in the shNC, and vice versa in the GSTM3-OE (Figure 8E). Totally, our superficial results indicated that GSTM3 might inhibit PC progression by sponging JAK-STAT, but further investigations were still needed.

## Discussion

Pancreatic cancer remains an extremely malignant tumor with a high mortality rate and dismal prognosis due to delayed diagnosis and early metastasis. The treatment of PDAC requires novel targets and increasing interest in research regarding novel cancer biomarkers has been focused on the GST family which contributes to the malignant phenotype of many cancers. GSTM3, a member of the mu class of the GST family, was related to the regulation of susceptibility to malignancies (20, 21). However, the role of GSTM3 in tumorigenesis remains largely unknown.

Recently, GSTM3 has been identified to exert an oncogenic or anti-tumor role in different cancers by targeting various signaling pathways. Wang et al. (22) showed that GSTM3 was associated with the susceptibility to renal cell carcinoma (RCC) and suppressed ROS activity and RCC progression. Similarly, Tan et al. (11) found that overexpression of GSTM3 reduced the anchorage-independent growth of RCC. Among patients with hepatic cancer, GSTM3 reversed radio-resistance and regulated the cell cycle/apoptosis (14). These data indicated that GSTM3 acted as a tumor suppressor. However, in colon cancer, Meding et al. (15) showed a strong association of GSTM3 expression with lymph node metastasis and cisplatin-treated metastatic colon cancer cells showed higher levels of GSTM3 than non-treated cells (23). A high GSTM3 expression was associated with worse survival among bladder cancer patients (24). For cervical cancer, Checa-Rojas et al. (16) pointed out that GSTM3 played a role in tumor progression through the MAPK and NF-κB pathways. Finally, Li et al. (25) revealed that downregulation of GSTM3 reduced the cellular proliferation in glioma. Thus, the biological role of GSTM3 appeared to be context dependent and might vary with different malignancies.

As far as we know, the associations between GSTM3 level and clinicopathological characteristics and prognosis of PDAC patients have not been investigated previously. Our results proved that a high level of GSTM3 was correlated with a good prognosis which implies its anti-tumor effect in PC. Furthermore, the effects of GSTM3 overexpression or knockdown on the proliferation and cell cycle of PC cells were evaluated using cell function assays and animal models. GSTM3 overexpression could inhibit cell proliferation by enhancing G0/G1 retention which also indicated that GSTM3 exerted a tumor suppressing role. In addition, the ROS level and glycolysis were assessed via transfection with shGSTM3 and GSTM3-OE. We revealed that overexpression of GSTM3 could inhibit the ROS level and reduce the level of glycolysis in PC cells, and the opposite outcome was presented in the knockdown group. RNA sequencing and western blot analysis revealed that GSTM3 might exert its role in PC partially through JAK-STAT signaling pathway. To some extent, our results showed that GSTM3 might exert a tumor suppressive role in PC and revealed candidate therapeutic targets in PDAC.

ROS are reactive species that originate from incomplete reduction of oxygen with high chemical reactivity, including the hydroxyl radical, superoxide anion, and hydrogen peroxide (26). ROS are considered damaging agents and are conducive to tumorigenesis by serving as mutagens and promoting genomic instability (27, 28). ROS could inhibit macromolecules such as lipids, nucleic acids, and proteins (29, 30). Accumulation of ROS led to the oxidative stress mediating pathology which was associated with the carcinogenesis (27). GSTs belong to the super family of phase II metabolic isozymes and protect cells against electrophilic damage by catalyzing ROS conjugation (31, 32). Our results showed that loss of GSTM3 in PC cells remarkably increased the levels of ROS. Elevated ROS exhibited a significant function in cancer cell viability and proliferation (33, 34). This finding was in accordance with the results of functional assay in our study, indicating that GSTM3 exerted anti-tumor effects in PC.

Decades ago, Otto Warburg put forward that cancer cells depended on glycolysis to support cellular growth even under normoxic conditions (the so-called “Warburg effect”) (35, 36). Tumor cells have a strong ability to survive in poorly nourishing environments by enhancing glycolysis (a process termed metabolic reprogramming) (37). Cellular metabolic reprogramming is regarded as one of the hallmarks of cancer cells and considered as the origin of cancer (38). PC cells have been proven to manifest extensive enhancement of glycolysis, including the overexpression of glycolytic enzymes and increased lactate production driven by cancer genes or the tumor microenvironment (39, 40). In our study, the GSTM3 silenced cells showed increased glycolysis and overexpression of GSTM3 decreased the level of glycolysis. Elevated glycolysis implied cancer progression in the GSTM3 silenced group, in keeping with the anti-tumor effect of GSTM3. The alteration of the glycolytic rate implied stress responses of metabolic reprogramming. The high-glycolysis phenotype of PC enhanced tumor progression by promoting epithelial mesenchymal transition, angiogenesis, and distant metastatic colonization (41). Moreover, glycolytic enzymes such as HK2, PKM2, and LDHB were overexpressed in PC (42–44). These were also detected in our study by western blot assay, further proving the alteration of glycolysis in PC tumorigeneses. The relationship between enhanced glycolysis and the oncogenic ability of PC could produce new therapeutic approaches targeting cancer metabolism. In addition, increased ROS could be generated as one of the by-products due to increased metabolic activities. And excessive accumulation of ROS in turn affect the metabolic reprogramming (45, 46). The relation between glycolysis and ROS in PC need to be further studied.

High-throughput screening performed by the gain or loss-of-function studies in PC cells showed that the differentially expressed genes was partially related to the metabolic process and antioxidant activity. And the KEGG pathway enrichment indicated several classical pathways such as JAK-STAT, cAMP, and PI3K-AKT. Further validation experiment proved JAK2-STAT4 was regulated by GSTM3 in PC. JAK/STAT was a common signal transduction pathway activated by many cytokines. STAT families were regarded as important components of DNA binding proteins that activate gene transcription (47). STAT4 might induce inflammation and promote tumor growth by regulating the immune response. Upon binding to IL12R, the JAK2 was linked to IL12Rβ2, and then STAT4 was phosphorylated, after which the dimerized STAT4 could be transferred to the nucleus to alter gene expression (48, 49). The results of sequencing were partially proven by western blot in our study, from which we might deduce that GSTM3 exerted a negative effect on PC progression via JAK-STAT. Other molecular mechanisms may also be involved, but await further investigation.

In conclusion, we found a correlation between the abundance of GSTM3 and better prognosis in patients and we believe that GSTM3 could be used as a predictor of prognosis. Our study also indicated that GSMT3 could inhibit PDAC cell proliferation and glycolysis. GSTM3 acted as a tumor suppressive factor and could potentially be an excellent candidate for target gene-based therapies for PC.

## Data Availability Statement

The original contributions presented in the study are publicly available. This data can be found here: https://www.ncbi.nlm.nih.gov/bioproject/PRJNA646321.

## Ethics Statement

The studies involving human participants were reviewed and approved by the Ethics Committee of Peking Union Medical College Hospital. The patients/participants provided their written informed consent to participate in this study. The animal study was reviewed and approved by Animal welfare and ethics committee from Peking Union Medical College Hospital.

## Author Contributions

YZ and MD designed concept of this manuscript. SW conducted the literature review and drafted the manuscript. LY reviewed the manuscript and made significant revisions on the drafts. SW and JY contributed to verify the data analysis. CD scrutinized the data. JL and SW prepared the pathological analysis. All authors approved the final version.

## Conflict of Interest

The authors declare that the research was conducted in the absence of any commercial or financial relationships that could be construed as a potential conflict of interest.
